# Counted, not just rescued: Nepal’s flood, and the test of an ‘Always On’ health system

**DOI:** 10.1371/journal.pgph.0007486

**Published:** 2026-10-15

**Authors:** Krishna Kumar Aryal, Anil Bhattarai, Akina Shrestha, Sirjana Adhikari, Kjell Arne Johansson

**Affiliations:** 1 Department of Global Public Health and Primary Care, Bergen Centre for Ethics and Priority Setting in Health (BCEPS), University of Bergen, Bergen, Norway; 2 Nepal Health Economics Association, Kathmandu, Nepal; 3 Department of Public Health, Kathmandu University School of Medical Sciences, Dhulikhel, Nepal; PLOS: Public Library of Science, UNITED STATES OF AMERICA

On 26 August 2026, an ice-and-rock avalanche on Nepal’s northern border with China, triggered a flash flood down the Bhotekoshi-Trishuli corridor. By 25 September, 13,795 people had been rescued, 1,453 deaths confirmed, 5,285 missing, and 10,988 required medical treatment [1]. The confirmed deaths alone already outrank seven of Nepal’s twenty-one tracked causes of death; if everyone missing is confirmed dead, this single event would fall within top ten leading causes of death in a typical year [2]. A flood is not usually counted as a burden-of-disease event. This one should be, and the health system’s response must be judged accordingly: across preparedness, response, and long-term resilience.

The health system was itself a casualty: four health facilities and two warehouses destroyed, three more damaged, and 19 health workers missing [3]. Thirty-five motorable and 45 suspension bridges were washed away, cutting off access to the nearest secondary level care in Nuwakot and Dhading district hospitals in already hard-to-reach terrain [1]. Within 24 hours, surveillance recorded acute gastroenteritis, influenza-like illness, and fever-with-rash cases at displacement sites, while a third of water samples tested were contaminated – early signs of the outbreak risk when water, sanitation, and referral systems fail together [4]. With thousands of people still in temporary shelters, the risk of further communicable disease outbreaks remains high.

Nepal’s health system has faced this test before – four times at a major scale in nine years: the 2015 Gorkha earthquake, the 2017 Terai floods, the 2023 Jajarkot earthquake, and the 2024 floods and landslides. Each time, facilities were damaged or cut off, referral routes lost, and immunisation and maternal services disrupted; none prompted rebuilding to a higher standard. This flood is a fifth, on the same fault lines (Fig 1).

**Fig 1:**
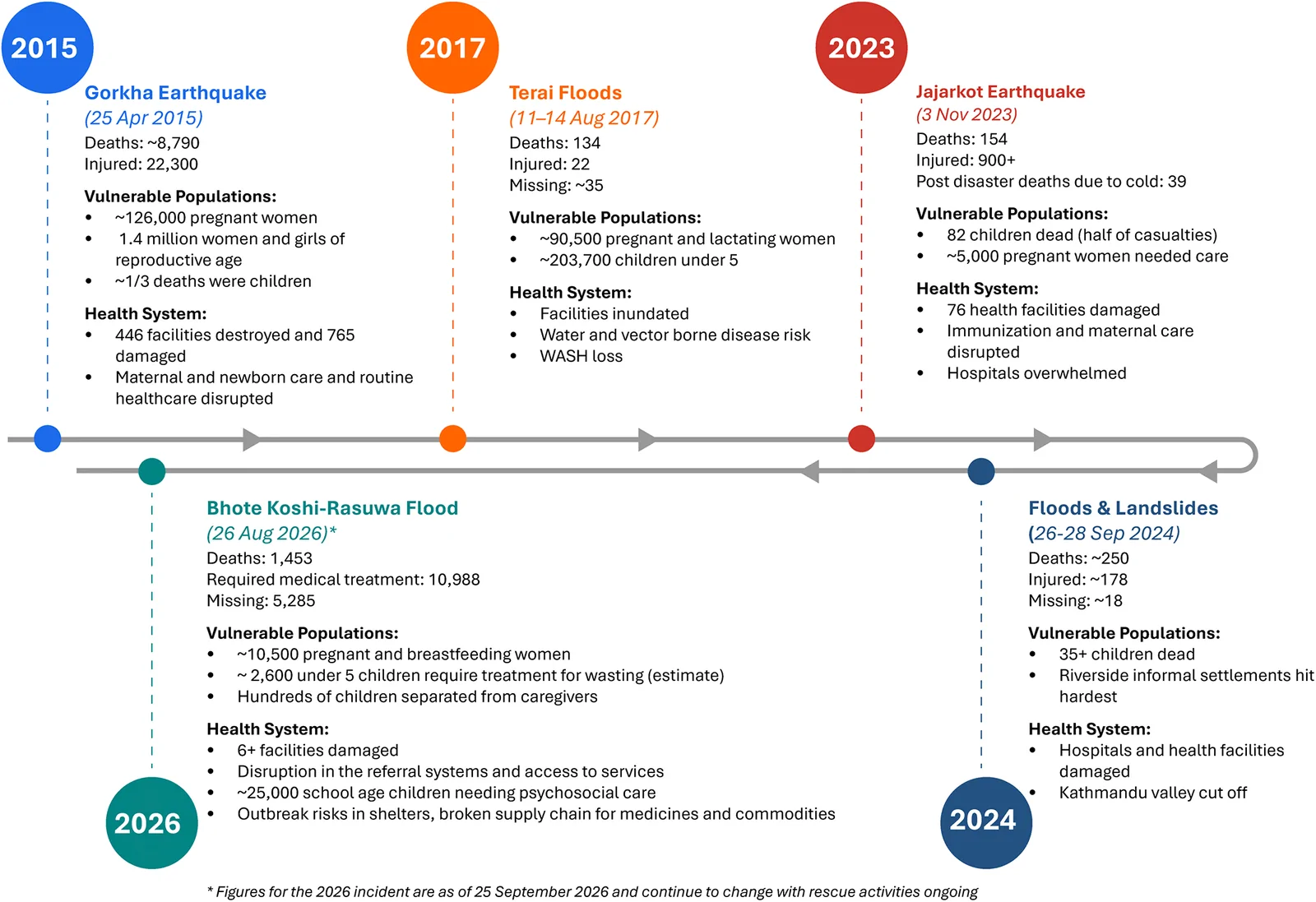
Major disasters in Nepal, 2015 to 2026: Health systems burden and other associated effects across five major rapid onset disasters. *Source: Authors’ compilation based on Government of Nepal assessments (National Planning Commission post disaster and post flood needs assessments, NDRRMA and Ministry of Home Affairs bulletins) and United Nations agency situation reports (OCHA, UNICEF, UNFPA and WHO), 2015–2026*.

Resilience is often measured by whether services nominally continued. Witter and colleagues’ 2023 reconceptualization reframes resilience around equity: not whether the system kept functioning, but who absorbed the strain of it doing so? [5] In this flood, that answer is already visible in who is left to cope without it: 19,284 households and 84,270 people were affected, including 43,085 women, 32,021 children and adolescents (7,048 under 5), 1,854 people with disabilities, and 8,603 elderly [6]. Resilience will be judged less by the emergency response than by what follows: how quickly facilities are rebuilt and routine maternal, newborn, and non-communicable disease care restored.

The fourth edition of Disease Control Priorities (DCP4) argues health systems should be “Always On” – standing capability built into routine care, not assembled after the fact – through dual-use infrastructure that scales rather than switches on [7]. Nepal has been building toward this since 2015: Health Emergency Operation Centres, a hub-and-satellite referral system, minimum service standards, oxygen and laboratory capacity expanded during COVID-19, and Emergency Medical Deployment Teams alongside community surveillance and hospital disaster plans. In a federal system, being “Always On” is as much a governance challenge as a technical one. Sustaining these capabilities require clear ownership, reliable financing and coordinated preparedness across all tiers of government. Nepal has taken steps through designated hub hospitals which hold standing disaster preparedness plans to coordinate emergency response and referrals with most incorporating provisions for surge capacity [8]. However, at the local level, legal responsibilities still exceed the capacity and resources available to fulfil them [9]. As a result, accountability for rebuilding and maintaining surge readiness remains fragmented, while funds and support often reach the front line slowly. The flood therefore tests not only the infrastructure, but also the effectiveness of intergovernmental fiscal transfer, institutional coordination, and shared responsibilities.

This flood is the live audit of that investment: did the destroyed health posts have any dual-use or surge design; did referral protocols anticipate losing 80 bridges at once? So far, the answer to both looks like “No”. Nepal is now finalising its national health benefits package through a systematic priority-setting exercise toward universal health coverage by 2030, raising a broader question: how should national priority-setting for health – including essential emergency and critical care [10] – build in disasters like this? That work, which several of the authors support at Nepal’s Ministry of Health and Food Safety, offers one avenue: building continuity of basic services, including EECC, into the revised basic health services package as a standing requirement, not an emergency add-on.

Rebuilding to disaster-resilient standards helps, but at this scale, is not enough: anything fixed in place can be swept away again. What must hold is the referral network actually activated rather than assumed, with strategic facilities resourced and ready; emergency transport that reaches patients in time to prevent avoidable deaths; and humanitarian aid that reaches frontline health workers, so they can return to work quickly. Routine care needs its own protection: after the 2015 earthquake, as the health system prioritised trauma care over routine services, admissions for maternal and other routine conditions at a Kathmandu tertiary hospital fell sharply and stayed low for months, showing a disaster’s harm reaches well beyond the injured [11]. These consequences extend beyond physical injuries to mental health. Following the 2015 earthquake, psychosocial needs increased sharply, but the response was largely improvised because mental health support had not been adequately integrated into emergency preparedness plans, a gap a standing system should close [12]. The health workers are among the affected too, with 19 missing in this flood, so safeguarding their wellbeing and swift return to work is itself part of keeping care on. Protecting the routine care is what keeps this flood from adding a second, quieter toll to Nepal’s burden of disease.

Although the final toll remains uncertain, deaths from this single event may rival those of major chronic conditions; if the missing are confirmed dead, this one flood will have killed nearly as many people in Nepal as diabetes does in a year (7,976 deaths in 2023) [2]. Matching that scale calls for the standing, all-hazard capacity that WHO’s disaster risk management framework urges and that DCP4 calls “Always On” [7,13]. This requires funding essential services, including EECC, through recurrent budget lines and regular fiscal transfers to provincial and local governments, ensuring readiness is maintained between disasters rather than rebuilt after each shock. Nepal’s own disaster law already mandates such funds across all three tiers of government, yet local resources and financial flexibility remain limited [9]. This is reinforced by the global experience as analysis of the COVID-19 response found that health security pursued in parallel to universal health coverage, rather than integrated with it, left systems fragmented and exposed, and that building preparedness into the routine systems and budgets that deliver everyday care is a more resilient course for low and middle income countries [14]. We echo the view of Karkey et al., published while this paper was being finalized, that health systems must be ready to absorb shocks of this magnitude while sustaining essential health services [15]. Financing that capacity as routine health spending, rather than mobilising it as relief after each disaster, is the policy test this flood now sets.
